# Comparison of Commonly Applied Outcome Inventories as Measures of General Internalizing Pathology in Psychological Therapies

**DOI:** 10.1002/cpp.70270

**Published:** 2026-04-15

**Authors:** Sakari J. Lintula, Suoma E. Saarni, Michael Barkham, Tom H. Rosenström

**Affiliations:** ^1^ Department of Psychology, Faculty of Medicine University of Helsinki Helsinki Finland; ^2^ HUS Helsinki University Hospital and University of Helsinki Helsinki Finland; ^3^ Tampere University Tampere Finland; ^4^ Päijät‐Häme Wellbeing County Lahti Finland; ^5^ School of Psychology University of Sheffield Sheffield UK

**Keywords:** CORE‐10, CORE‐OM, OASIS, PHQ‐9

## Abstract

Measuring general factors to capture severity of psychopathology broadly can be useful for assessing treatment outcomes of psychological therapies. Namely, general factors are suitable for broadband measurement, which has become increasingly important due to efforts to standardize outcome measurement. Reasons for measuring general factors include their predictive validity, relatability to parallel research in epidemiology, genetics and neurobiology, simplicity due to unidimensionality, longitudinal structural validity and potential for capturing transdiagnostic change. Psychotherapy register data (*N* = 5223; 4059, 77.7%, identify as female) of Finnish speaking adult patients entering psychological therapy were used to compare three patient self‐report inventories suggested for routine use—Patient Health Questionnaire‐9 (PHQ‐9), Clinical Outcomes in Routine Evaluation–Outcome Measure (CORE‐OM) and CORE‐10—as measures of a general internalizing pathology factor. A multidimensional factor model was estimated, and noise factors were marginalized out of the analysis. Fisher information about internalizing was computed for each inventory. CORE‐OM had high total Fisher information about internalizing, but per item information was markedly lower compared to CORE‐10. Surprisingly, though not included a priori in the set of compared inventories, the Overall Anxiety Severity and Impairment Scale (OASIS) was as effective as CORE‐10 per item, though OASIS provided low total information due to only comprising five items and was confounded by an anxiety specific factor. PHQ‐9 did not perform well as a measure of internalizing. Results suggested CORE‐10 to be a valid, brief, option for broadband outcome measurement in psychological therapies.

## Summary

- The CORE‐10 is an efficient option for transdiagnostic outcome monitoring, compared to PHQ‐9 or CORE‐OM. It is freely available with multiple translations.
- Longer measures do not guarantee greater clinical utility; per item gains in outcome measurement reliability decrease by addition of suboptimal items. CORE‐OM is most reliable, but per item is not as effective as CORE‐10.
- Brief diagnosis specific measures (e.g., OASIS) may be useful primarily in combination with transdiagnostic outcome monitoring.
- Appropriate comparisons of inventories in a general‐purpose role requires identification their shared marginal factor(s).

Outcome measurement in psychological therapies can rely on diagnostic‐group specific inventories (Farber, Gage, and Kemmer 2023) or broadband inventories (Barkham 2021). Growing evidence suggests that broadband measurement might have potential over the diagnostic specific approach: Specifically, a general factor–based measurement model might be a simple, yet valid, alternative. In the present article, we analyse common outcome inventories in this framework. Specifically, we inspect which commonly applied inventories might best capture general factors.

Despite the ongoing debate (Pettersson 2025), structural analyses repeatedly suggest there are underlying transdiagnostic general factors^1^ of mental disorders (Forbes et al. 2023; Kotov et al. 2017; Wright et al. 2025). Such factors include the ‘p’ factor (Caspi et al. 2024; Lahey et al. 2021), general externalizing (Achenbach 1966; Krueger et al. 2005) and internalizing pathology factors (Achenbach 1966; Wright et al. 2025).

Measuring such general factors is promising (see also Pettersson 2023). They often predict outcomes better than diagnostic groups, are simpler for standardized use, have longitudinal structural validity and might confound disorders and help explain symptom persistence (Eaton et al. 2013; Gluschkoff et al. 2019; Kim et al. 2021; Pettersson et al. 2018). General factor research aligns with contemporaneous genetic and neuroimaging studies (Allegrini et al. 2020; Durham et al. 2021; Krueger 2025). Finally, psychological treatment tends to have broad, observed, effects (Barlow et al. 2014; Wampold 2015).

Specifically, a general internalizing pathology factor might be most relevant in psychological therapies for common mental disorders, as the latter comprises internalizing, not externalizing, problems. However, the p factor has been observed to be closely associated with personality disorders (Gluschkoff et al. 2019; Smith et al. 2020). Thus, it might also have merit as a measured construct in psychological therapies. Further, in hierarchical models, internalizing disorders would always contain variation of p as well (Kotov et al. 2017; Lahey et al. 2021).

The current study makes three additions to the literature. First, in any analysis of any general factor, observations inevitably contain variation attributable both to a general factor (e.g., internalizing) and specific factors (e.g., anxiety). This prevents analysis of a general factor in isolation (Stucky et al. 2013). Developments in IRT now provide a method for marginalizing out the specific factors, which allows isolating a general factor (Ip 2010). To our knowledge, there are no studies using this isolate‐and‐analyse approach in psychological therapy outcome measure development. We use this method and provide tools for future application. Second, most psychometric analyses have evaluated inventories individually instead of jointly (Kocalevent et al. 2013; Kroenke et al. 2001; Zeldovich and Alexandrowicz 2019). This makes separating a general factor from specific factors more difficult. We conduct a joint analysis of different inventories. Third, as the main addition to the literature, we compare commonly applied outcome measures already implemented in psychological therapy practice internationally as measures of a general factor.

Three self‐report inventories that have been proposed for standardized use in psychological therapies are compared as measures of a general factor: the Clinical Outcomes in Routine Evaluation–Outcome Measure (CORE‐OM; Barkham et al. 2006; Evans et al. 2002), CORE‐10 (Barkham et al. 2013) and the Patient Health Questionnaire‐9 (PHQ‐9; Kroenke et al. 2001). CORE‐OM and CORE‐10 have been suggested for broadband outcome measurement. PHQ‐9, alongside a generalized anxiety disorder inventory (GAD‐7; Spitzer et al. 2006), has been suggested as a way to achieve parsimonious standardized outcome measurement for anxiety and depression (Farber, Gage, Kemmer, and White 2023; Obbarius et al. 2017), and using this approach has been mandated by institutional funding policies (Farber and Kemmer 2020).

Our aim is, first, to estimate and interpret a general factor model. We expect a general internalizing pathology factor to emerge as the study population mostly has internalizing problems, for example, depression and anxiety disorders. Second, we quantify which of the three inventories is most informative about the estimated general factor in the context of common mental disorders.

## 1 Methods

### 1.1 Sample

Data were obtained from the Finnish Psychotherapy Quality Registry (FPQR), a routine clinical registry covering all outsourced psychotherapies and part of in‐house services in Helsinki University Hospital HUS region between June 2018 and September 2023 (Saarni et al. 2023). Of an initial 5569 entries of 18 years, or older, patients, data preprocessing included removal of 135 entries with missing identification number. Thus, 5434 adult patients were included; 5223 fully completed all baseline questionnaires and 3099 end‐of‐treatment questionnaires. The missing end‐of‐treatment data were largely due to ongoing treatments at the time of data extraction. Patients complete their questionnaires digitally either by themselves or with their therapist's assistance. The measures used in the register were selected in an iterative consensus process by a national consortium of clinical experts during 2016–2018 (Saarni et al. 2023). Table 1 presents the distributions of background variables as well as descriptives of the outcome inventories. As seen in Table 1, a total of 96.0% of the patients had an anxiety or mood disorder diagnosis set by a physician according to ICD‐10 (International Classification of Diseases 10th edition).

**TABLE 1:** Descriptive statistics of adult patients in the Finnish Psychotherapy Quality Register.

	Registered to pretreatment assessment	Full pretreatment assessment^a^ available	Full pretreatment and posttreatment assessment^a^ available
	(*N* = 5434)	(*N* = 5223, 96.1%)	(*N* = 3099, 58.0%)
	*n* (%) or *M* (SD)	*n* (%) or *M* (SD)	*n* (%) or *M* (SD)
Gender			
Female	4222 (77.7)	4059 (77.7)	2434 (78.5)
Male	1207 (22.2)	1160 (22.3)	662 (21.4)
Other	< 5 (< 0.1)^b^	< 5 (< 0.1)^b^	< 5 (< 0.1)^b^
Missing	< 5 (< 0.1)^b^	< 5 (< 0.1)^b^	< 5 (< 0.1)^b^
Age			
Years	38.1 (13.7)	38.3 (13.8)	38.2 (13.8)
Occupational status			
Employed	2241 (41.2)	2188 (41.9)	1287 (41.5)
Student	655 (12.1)	637 (12.2)	385 (12.4)
Unemployed	547 (10.1)	537 (10.3)	335 (10.8)
Stay‐at‐home parent	299 (5.5)	294 (5.6)	168 (5.4)
Retiree	296 (5.5)	286 (5.5)	175 (5.6)
Rehab. or sickness allowance or disability pension	720 (13.3)	706 (13.6)	427 (13.8)
Other	448 (8.2)	442 (8.5)	274 (8.8)
Missing	228 (4.2)	134 (2.6)	48 (1.5)
Psych. medication			
Concurrent	2582 (47.5)	2518 (48.2)	1504 (48.5)
Past	966 (17.8)	947 (18.1)	563 (18.2)
Never	1658 (30.5)	1624 (31.1)	984 (31.8)
Missing	228 (4.2)	134 (2.6)	48 (1.5)
Primary ICD‐10 Diagnosis			
Mood F30–39	2498 (46.0)	2402 (46.0)	1442 (46.5)
Anxiety F40–48	2606 (48.0)	2512 (48.1)	1472 (47.5)
Psychotic F20–29	69 (1.3)	65 (1.2)	42 (1.4)
Other	260 (4.8)	244 (4.7)	143 (4.6)
Missing	1 (< 0.1)	0	0
Measure^c^			
CORE‐OM	NA	50.8 (20.0)	38.1 (21.6)
CORE‐10		16.0 (6.8)	11.9 (7.3)
PHQ‐9		9.9 (5.6)	7.0 (5.5)
OASIS		9.5 (3.9)	7.1 (4.2)
AUDIT‐C		2.4 (2.1)	2.3 (2.0)

^a^ Complete response pattern to CORE‐OM, PHQ‐9, OASIS and AUDIT‐C.

^b^ Under Finnish registry data scientific publication disclosure (Findata), reporting small cell counts is prohibited, and, thus, some gender counts are suppressed to strengthen anonymity.

^c^ Sum scores of all items in the inventory are shown to describe the data, but note that they are not recommended for outcome evaluation practice.

### 1.2 Variables

We chose the five inventories (PHQ‐9, OASIS, CORE‐OM, CORE‐10 and AUDIT‐C) that are used for all adult patients in the FPQR registry, selected to obtain as wide as possible view of mental health conditions. Overall, the obtained item set in the FPQR leans towards the internalizing spectrum, as the measures are intended for outcome measurement in psychotherapy, which is most applied to internalizing, not externalizing, problems. The item set does include items related to aggression and alcohol misuse, that is, externalizing problems. This helps identify what kind of variation the estimated general factor captures. In this respect, we had the opportunity to inspect if the estimated general factor had a factor structure that aligns with the p factor (the estimated general factor is associated with all items) or internalizing (not associated with externalizing items).

CORE‐OM (Evans et al. 2002; Honkalampi et al. 2017) comprises 34 items about a person's level of psychological distress. The items refer to experiences during the past week on a 5‐point scale with anchor points of 0 (*not at all*) to 4 (*most or all of the time*). CORE‐OM contains items related to mood and anxiety, such as hopelessness, constant sadness and anxiety, as well as items denoting “risk” to self and risk to others (e.g., aggressive behaviour). As CORE‐OM item 22 (‘I have threatened or intimidated another person’) had only two observations for ‘most or all of the time’, they were truncated to value 3. For an independent review of psychometric properties of CORE‐OM, see Zeldovich and Alexandrowicz (2019).

CORE‐10 (Barkham et al. 2013) comprised a subset of 10 items within the CORE‐OM. In the current study, these 10 items were drawn from CORE‐OM to construct the CORE‐10 rather than asking participants to complete the items again. Hence, CORE‐10 items were derived from the embedded items within CORE‐OM rather than being administered separately. They were ordered in the same sequence they appeared in the CORE‐OM with the same scoring and time frame. Hereon, we refer to CORE‐10 items embedded within CORE‐OM as CORE‐10, for brevity. Note that items of CORE‐10 were not administered twice nor duplicated in any factor analysis.

PHQ‐9 (Kroenke et al. 2001) comprises nine items focusing on a person's experience of the symptoms of depression and map onto the DSM criteria. The time frame is the past 2 weeks with items being scored on an ordered scale from 0 (*not at all*) to 3 (*nearly every day*). For basic psychometric properties and validity of PHQ‐9, see Kocalevent et al. (2013).

Overall Anxiety Severity and Impairment Scale (OASIS) contains five items. In OASIS, the patient responds to five statements about their mental well‐being during the past week on an ordered scale from 0 (*not once*) to 4 (*all of the time*). For basic psychometric properties and validity of OASIS, see Bragdon et al. (2016) and Campbell‐Sills et al. (2009). Though not, to our knowledge, suggested as a candidate for standardized use in outcome measurement, OASIS is included to obtain a wider range of symptomatology in the total item set.

Alcohol Use Disorder Identification Test (AUDIT) (Bush et al. 1998) contains 10 items. We used the three item AUDIT‐C (Bush et al. 1998). AUDIT and AUDIT‐C contain items related to alcohol risk behaviour. In AUDIT‐C items, the patient responds to statements about their alcohol use frequency and typical amount of consumption on an ordered scale of 0 (*never*) to 4 (*daily or almost daily*).

The Finnish versions of all self‐report measures were used. In total, they yielded an item pool of 51 *unique* items (i.e., we did not double count the CORE‐10 items but instead used those already reported as a part of the CORE‐OM).

### 1.3 Statistical Methods

Our statistical procedures followed five stages. First, we quantified how much variance–covariance of the item pool the first principal component explained. This is a common way to approximate how ‘large’ the first factor would be. A polychoric correlation matrix was used. Second, we conducted a parallel analysis (Garrido et al. 2013; Horn 1965) to decide on the number of factors in the data. The above steps were done first using pretreatment cross‐sectional observations and then with change scores. Change scores were computed as the difference between the pretreatment and the posttreatment item scores. Analysis of change scores was done to verify that the large share of variance–covariance explained by the first component is not specific to cross‐sectional observations (i.e., a main component is relevant for monitoring within‐patient changes too, not just between‐patient differences). Parallel analysis of the change scores is given for completeness.

Third, we conducted an Exploratory Factor Analysis (EFA) with bi‐geomin rotation on the pretreatment item pool to obtain a factor structure with a general factor (Jennrich and Bentler 2012). Note that we expected a general internalizing pathology factor to emerge but, simultaneously, had the opportunity to evaluate if, instead, a p‐consistent factor would emerge. Thus, EFA was used instead of confirmatory factor analysis.

Fourth, as the data are inevitably multidimensional, we used a procedure to isolate the estimated general factor from the specific factors, conceptually speaking. Mathematically, we marginalized over the specific factors and obtained marginal parameter estimates for the estimated general factor (Ip 2010). An overview and an R‐script are provided for the marginalization in Section S2.

For psychometric comprehensiveness, we also provide longitudinal measurement invariance modelling results (Liu et al. 2017; Rosenström et al. 2022). All available observations were used to estimate the polychoric correlations using pairwise complete data for the parallel analyses and in the EFA.

Fifth, we computed the (marginal) Fisher information from the EFA model's general factor parameters (Krueger and Finger 2001). Note that Fisher information is referred as information function, test information or item information in IRT literature (Lord et al. 1968; Markon 2013). We specify that the information used is Fisher information, not Shannon information (Shannon 1948) nor information in the colloquial sense of knowledge. The following Fisher information–based statistics were computed:

- Point item information is the Fisher information about the estimated general factor for a single item on a single point (i.e., location or level) of the estimated general factor. Therefore, it varies pointwise depending on the point at which it is computed.
- Point test information is the sum of point item information of all items in a measure. It gives the Fisher information about the estimated general factor for the entire set of items at a single point of the factor.
- Total item, or test, information is defined as the area under the point item, or test, information curve. It summarizes Fisher information over the entire range of the estimated general factor as a single value.^2^

We computed point information and total information for all items and measures. Point item, or point test, information is called simply item, or test, information in IRT. We specifically call them point‐wise statistics to clearly distinguish them from the total item, or test, information statistics. To account for measure length, total test information was divided by the number of items in the respective measure.

Information estimates were also transformed to standard error of measurement: $\text{StdEM}=\frac{1}{\sqrt{I}}$, where I is any point‐wise information statistic outlined above. Inspection of the point test information for all the measures, and of StdEM computed from it, was done to compare the measures with respect to their coverage of the estimated general factor.

For the statistics of main interest, nonparametric bootstrap 95% percentile confidence intervals were computed using 1000 resamples. For openness, reproducibility and for a more detailed account of the analysis, statistical methods are comprehensively described in Section S1. The marginalization procedure is described in Section S2. For all multivariate analysis, all items of all measures were used to estimate the estimated general factor. CORE‐10 items overlapping with CORE‐OM, although they can be considered as elements of either inventory, were not duplicated nor treated differentially compared to the other items in any analysis. All statistical analysis and visualization were done using R software Version 4.4.1. For the EFAs, the *lavaan* R‐package Version 0.6–17 (Rosseel 2012) was used. The *ggplot2* R‐package (Wickham 2011) was used for all visualizations. A sensitivity analysis was conducted by excluding items of CORE‐OM not in CORE‐10 from the EFA.

Generative AI was used to check grammar.

## 2 Results

### 2.1 Covariance Explained, Marginalization and Interpretation of the Estimated General Factor

**FIGURE 1:**
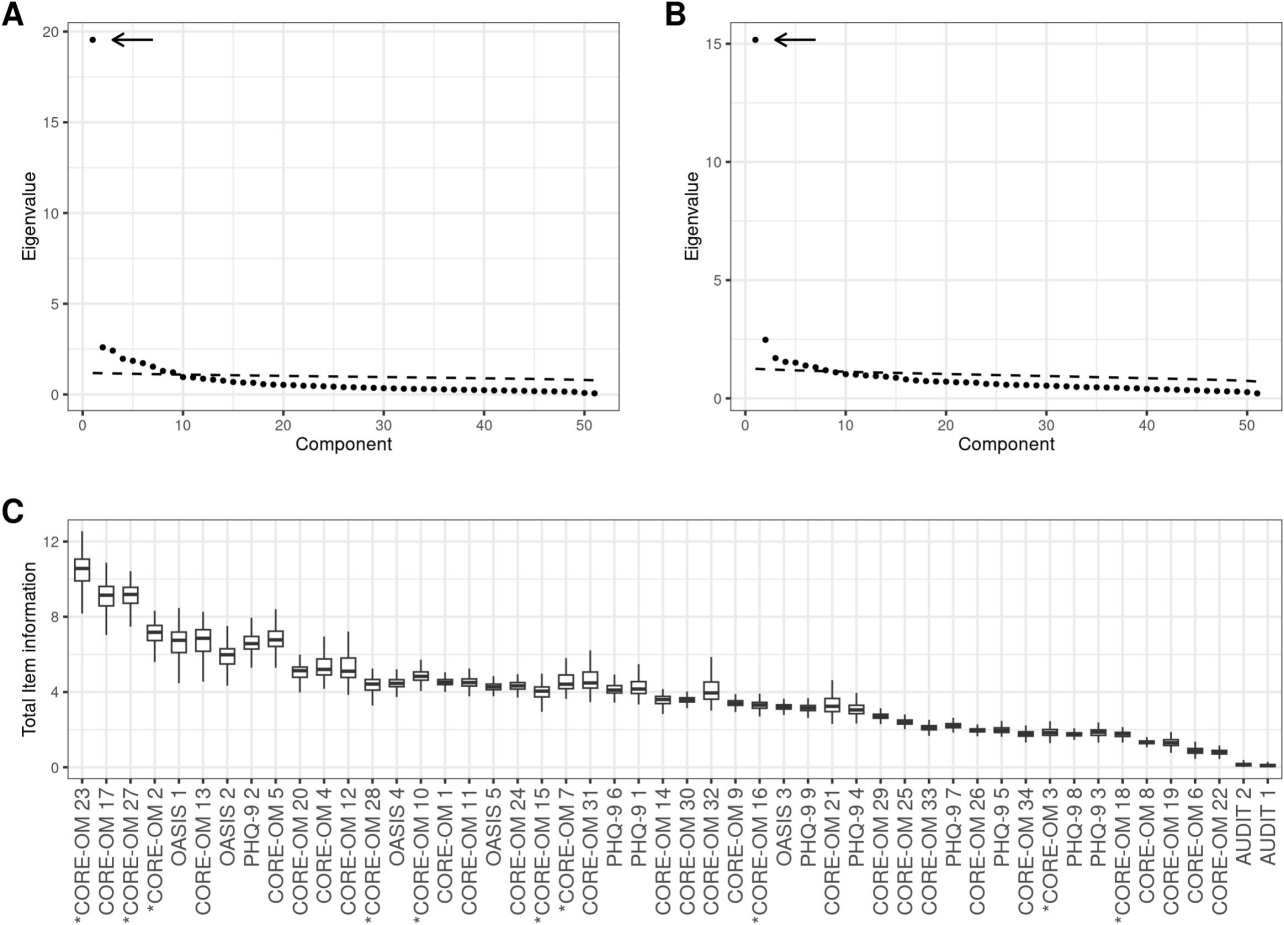
(A) Scree plot and parallel analysis of the polychoric correlation matrix of pretreatment items. Dashed line is parallel analysis criterion for choosing the number of factors to retain. The arrow emphasizes the first component. (B) As in (A) but for the item change scores. (C) Total item information about the general factor for each item with Tukey's boxplots of the bootstrapped sample distribution. Total item information summarizes how much information overall an item gives about the general factor. Items with an asterisk (*) are CORE‐10 items.

The first principal component explained 38.2% of all covariance in the pretreatment observations, which was 7.3 times greater than the covariance explained by the second principal component, as is shown in Figure 1A. Covariance explained by subsequent principal components is summarized in Table 2. A similar finding was observed for the change‐score: The 1st component explained 29.7%.

**TABLE 2:** Explained variance of components.

	Explained variance (%)		
Component	Preassessment		Change score
1st	38.2		29.7
2nd	5.2		4.8
3rd	4.9		3.3
Sum of 4th to 9/8th^a^	19.3		13.7

^a^ The maximum number of factors suggested by parallel analysis was 9 and 8 for preassessment and change score, respectively.

The parallel analysis suggested nine factors, so we used a bi‐geomin rotated nine‐factor EFA. AUDIT‐3 item had extreme and instable parameter estimates, which were associated with nonconvergence in estimation. When comparing a nonconvergent EFA to EFA with AUDIT‐3 omitted, the difference in loadings patterns was small and in neither case did any AUDIT‐item load onto the general factor. Thus, we continued by omitting AUDIT‐3 from the EFA analysis. For detailed model parameters and fit, see Tables S1–S3.

The items (related to alcohol misuse) of AUDIT‐C, aggression items of CORE‐OM (Items 6 and 22) did not load on to the general factor. Thus, because the estimated general factor was not observed to associate with any aggression and alcohol misuse items, it was interpreted as a general internalizing factor, not as the p factor. The remaining items loaded on to the general factor, which explained most (57.6%) of covariance explained by all factors.

Notably, isolating the general factor via marginalization sometimes halved the factor loadings. This suggests that marginalization was necessary to obtain valid estimates for subsequent analysis.

Longitudinal measurement invariance modelling supported the use of all measures over the course of psychological therapy, as no clear violations of invariance were seen—see Table S4.

### 2.2 Fisher Information–Based Comparisons of Outcome Inventories

**FIGURE 2:**
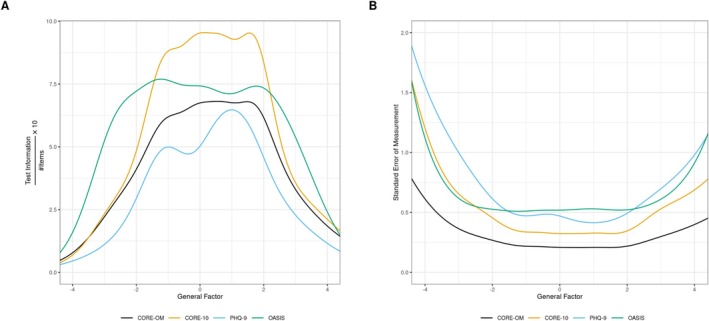
(A) Averaged point test information curves, scaled to a 10‐item set $10\times \frac{I}{\#\text{items}}$. (B) Standard error of measurement computed from nonaveraged point test information curves.

For all 51 items, total test information was 199.4, which indicates overall Fisher information available about the estimated general factor. Three CORE‐OM items had larger total item information than the rest, as seen in Figure 1C: CORE‐OM 23, CORE‐OM 27 and CORE‐OM 17. The items CORE‐OM 23 and CORE‐OM 27 are also CORE‐10 items. Otherwise, Table 3 shows the computed total test information for each inventory and comparisons by averaged total test information. In Figure 2A,B, averaged point test information distribution and StdEM are shown, respectively. Though not in the a priori defined list of inventories to be compared, OASIS items had comparatively high Fisher information, and thus, OASIS was kept for subsequent analyses as well.

**TABLE 3:** Total test information estimates and differences ordered by average total test information.

Measure (number of items)	Total test information	95% CI	Averaged total test information	95% CI	Comparison	Difference in averaged information	95% CI
1. OASIS (5)	26.2	(20.2, 27.6)	5.24	(4.03, 5.51)	—		
2. CORE‐10 (10)	51.6	(45.5, 54.3)	5.16	(4.55, 5.43)	1. vs. 2.	0.08	(−0.77, 0.41)
3. CORE‐OM (34)	138.6	(131.5, 148.7)	4.08	(3.87, 4.37)	2. vs. 3.	1.08	(0.60, 1.16)
4. PHQ‐9 (9)	27.1	(26.0, 34.2)	3.01	(2.89, 3.80)	3. vs. 4.	1.07	(0.14, 1.29)

*Note:* All 95% CIs are from nonparametric percentile bootstrap with 1000 replications.

CORE‐OM had the highest total test information, but after averaging over number of items, it had the second lowest of the four inventories. As the longest measure, it had the best StdEM over the midrange from −2 to 2 of the estimated general factors (approximately 95% inner quantile of a standard normal distribution). CORE‐10 and OASIS had the highest averaged total test information of the existing measures (difference not significant; Table 3). It was second to CORE‐OM in StdEM, when not adjusting for number of items. PHQ‐9 had the lowest averaged total test information. It had a larger StdEM than CORE‐10 and CORE‐OM over the midrange. Only OASIS had a larger StdEM than PHQ‐9 over the midrange. OASIS performed unexpectedly well when adjusting for number of items. However, it lacked unadjusted test (total or point) information.

### 2.3 A Sensitivity Analysis for Inventory Length

Because CORE‐OM had 34 items, accounting for two thirds of the unique item pool (66.7%), we conducted a sensitivity analysis in which only the CORE‐10 items were included; that is, CORE‐OM items that were not in CORE‐10 were excluded. The result showed similar item information and total test information patterns as the main analysis, suggesting that the main analysis was not an artefact created by CORE‐OM comprising the most items in the whole item set. Total information estimates of the sensitivity analysis are given in Section S5.

## 3 Discussion

In this study, we estimated Fisher information about an estimated general factor for outcome measures using a large register sample of psychological therapies. To our knowledge, this is the first comparison of Fisher information estimates about an estimated general factor while marginalizing out specific factors in the context of psychological therapy outcome measurement. The factor structure indicated that the estimated general factor was interpretable as a general internalizing pathology factor (Achenbach 1966). CORE‐10 was arguably the best performing measure of the estimated internalizing factor.

The large amount of overlap within the item set suggests that a general factor could capture a substantial amount of all available variation in the routine outcome data. Along with other research outlined in the introduction (Pettersson 2023), the observed overlap supports the use of a broadband general factor–based measurement model. Furthermore, CORE‐10 is well suited for assessing such a broadband construct, as it performed best when adjusting for number of items while also being informative in total. In routine outcome measurement (Barkham et al. 2023; Boswell et al. 2015), burdening patients and therapists with lengthy questionnaires can be a problem (Saarni et al. 2023). The brevity of CORE‐10 might, therefore, be valuable, whereas CORE‐OM might be considered lengthy with respect to per item information yield, especially if used session by session.

The current article adds to an increasing body of literature focused on the validity and clinical utility of the CORE‐10, which has been found to be a viable routine outcome measure in multiple different settings and analyses (la Tona et al. 2023; Rosenström et al. 2022; Vos et al. 2026). This evidence is underpinned by the recommendation that outcome measures need to capture a greater bandwidth of patient experiences than is achieved by mono‐symptomatic measures of, for example, depression or anxiety separately (see Barkham et al. 2025). Consistent with this is the increasingly frequent recommendation for adopting a transdiagnostic approach to clinical assessment and practice, which would support the use of a more generic assessment measure (e.g., CORE‐OM) paired with an associated brief routine outcome monitoring (ROM) measure (e.g., CORE‐10). This approach has been recently adopted, for example, in Ecuador (Valdiviezo‐Oña et al. 2024). CORE‐10 is Fisher information‐wise effective, brief, transdiagnostic (as opposed to mono‐symptomatic) and a longitudinally measurement invariant (Rosenström et al. 2022) outcome inventory that is accumulating data on patient responses to psychological therapy internationally (Meglio et al. 2026). Thus, CORE‐10 is a valuable addition to the available tools for practitioners to adopt in their clinics.

Our results align with recent research on CORE‐10, suggesting that it captures broad, transdiagnostic, variations in patient symptomatology (Vos et al. 2026). CORE‐10 provides (Fisher) information effectively, when the intention is to measure transdiagnostic factors and associate them with external outcomes, for example. This can be valuable for the growing body of research, where the aim is modelling psychopathology and its outcomes (Krueger 2025).

Though not usually promoted for standardized use, OASIS items contained much Fisher information about internalizing. OASIS might not, however, be suitable for widespread implementation as a standalone measure because it provided limited total information due to the small number of items. Second, it was strongly loaded on to an anxiety‐specific factor and, thus, would not distinguish general internalizing pathology from anxiety specific pathology well for patients with high anxiety. However, given the small number of items of OASIS (i.e., 5) and the ability to discriminate in both the low and the high ends of internalizing, OASIS might be useful if used in combination with another inventory or multiple inventories.

As an example, PHQ‐9 has been suggested for standardized use alongside another anxiety inventory (Farber, Gage, and Kemmer 2023; Farber and Kemmer 2020). However, in line with recent research focused on structure of mental disorders (Forbes et al. 2023; Kotov et al. 2015), no clear depression factor, which PHQ‐9 might be expected to measure specifically (Kocalevent et al. 2013; Obbarius et al. 2017), emerged in our analysis (Table S2). Instead, PHQ‐9 largely loaded onto the internalizing factor (Section S4 and Table S2) and had a multidimensional specific factor structure—similarly to CORE‐OM and CORE‐10. Nevertheless, PHQ‐9 did not perform as well in terms of information about a general internalizing pathology as the other inventories.

### 3.1 Limitations

A limitation in the current study was that CORE‐10 was administered as a subset of CORE‐OM, meaning that results might not directly match onto a situation where CORE‐10 is solely administered. However, we have no reason to believe that if delivered as a stand‐alone inventory, the items would behave sufficiently differently to change the result reported here. Second, repeating these analyses with a different set of (or more) inventories would be useful to inspect how dependent the conducted analysis is on the set of inventories. Third, bifactor models are sometimes unstable in their parameter estimates. To some extent, instability was seen as skewed bootstrapped confidence intervals (Table S1).

## 4 Conclusions

When compared as a measure of a general factor of common mental disorders, the results suggest that CORE‐10 can be a valid option for brief outcome measurement in psychological therapies. Joint analyses with multiple inventories using latest methodology can provide more nuance to interpretations of existing measures.

## Funding

This work was supported by the Academy of Finland (334057, 335901 and 358138), the Finnish Social Security Institute (140/331/2021), NextGenerationEU and Finska Läkaresällskapet.

## Ethics Statement

Access to pseudonymized data for research purposes was based on an approval from a regional committee on research ethics (Helsinki University Hospital district, Approval ID: HUS/3150/2020). Because the study used a registry sample, informed consent was not required according to national laws.

## Conflicts of Interest

The authors declare no conflicts of interest.

## Supporting information


**Table S1:** General Factor parameters and total item information
**Table S2:** All standardized (unmarginalized) factor loadings of exploratory factor analysis with 9 factors.
**Table S3:** Factor correlations in explorative factor analysis.
**Table S4:** Longitudinal measurement invariance testing for CORE‐10, PHQ‐9 & OASIS.
**Table S5:** Total item information when omitting CORE‐OM items not included in CORE‐10.

## Data Availability

The data are not openly distributable due to Finnish law on the secondary use of personal data in social and health care. Reproducible code used for the analysis is available from an online resource; see Section S3. Otherwise, all analysis code is available upon request from the first author. We report how we determined our sample size, all data exclusions, all manipulations and all measures in the study. This study's design and its analysis were not preregistered.

## Endnotes

^1^
A general factor of psychopathology sometimes refers specifically to the ‘p’ factor (Caspi et al. 2024), but we use the term general factor in its literal sense: A factor that has generality due to a variety of mental health symptoms loading on it. This is to highlight the contrast to a specific factor, by which we mean a factor associated with a single mental health syndrome.

^2^
At least two alternative ways should be mentioned. First, test information utility, which is the Kullback–Leibler divergence between the posterior distribution of the latent factor, given the item response, and the prior distribution of the latent factor (Markon 2013). Second, expected Fisher information could be used, though it devalues Fisher information in the extreme ends under a normally distributed latent factor. We use the area under the Fisher information curve because it is simple and sufficient for comparing the measures.
