# Supplementary material for: Comparison of Commonly Applied Outcome Inventories as Measures of General Internalizing Pathology in Psychological Therapies
Source: Clin Psychol Psychother. 2026 Apr 15;33(2):e70270. doi: 10.1002/cpp.70270 (PMC13080418; doi:10.1002/cpp.70270)
Supplement: Supplementary file 1 — Table S1: General Factor parameters and total item information Table S2: All standardized (unmarginalized) factor loadings of exploratory factor analysis with 9 factors. Table S3: Factor correlations in explorative factor analysis. Table S4: Longitudinal measurement invariance testing for CORE‐10, PHQ‐9 & OASIS. Table S5: Total item information when omitting CORE‐OM items not included in CORE‐10. [file CPP-33-e70270-s001.docx]

## Section 1: Details and further rationale for the statistical analysis

The first aim of this study was to estimate and describe a general factor in the 51-item pool. To do this, we inspected the scree plot and eigenvalues of the polychoric correlation matrix of the pre-treatment variables. Eigenvalues of a correlation matrix indicate what proportion of variance-covariance is explained by an extracted component. A 51 -item correlation matrix has 51 eigenvalues, which sum to a total of 51. Hence, an eigenvalue of a correlation matrix, say, the first component explains $\frac{e_{1}}{51}$ of all (standardized) variance-covariance (correlation) where $e_{1}$ is the eigenvalue. We conducted a parallel analysis (Horn, 1965) to guide choosing the number of specific factors. Parallel analysis gives a criterion for the number of factors to retain by comparing the eigenvalues of the observed correlation matrix with those obtained from randomly generated correlation matrix thus correcting for sampling error. Note, that parallel analysis itself is not ‘designed’ for factors, but remains one of the most competitive methods for selecting the number of factors to retain (Lim & Jahng, 2019).

To further assess how well symptom (item) changes during psychological therapy can be attributed to the estimated general factor, first, we calculated change scores by subtracting pre- from post-treatment assessment for each item and inspected the ensuing scree plot and eigenvalues of the change-score (up- and downward category transitions) polychoric correlation matrix. Another parallel analysis was conducted for the change-score polychoric correlation matrix. I.e., we would expected there to be a large firsts component in, both, the ordinary polychoric correlation matrix as well as the change score polychoric correlation matrix, if a large general factor that could summarize variations in the outcome items well was present in the data.

To obtain information estimates, exploratory Factor Analysis (EFA) was estimated with bi-geomin rotation (Jennrich & Bentler, 2012). We used the ‘*lavaan’* -package version 0.6-17 (Rosseel, 2012) and a weighted least squares mean and variance corrected estimator with pairwise deletion while treating all variables as ordinal. Pairwise deletion uses all observations available in computing each correlation estimate. Scaled fit indices, as provided in lavaan, root mean squared error of approximation (RMSEA), comparative fit index (CFI), Tucker and Lewis’ index (TLI) and standardized root mean squared residual (SRMR), interpreted using the common cut-offs of RMSEA < 0.060, CFI > 0.950, TLI > 0.950 and SRMR < 0.060. Note that model fit was not of interest, since we would anyhow obtain as many specific factors as necessary. All specific factors would later on be “marginalized out” from the analysis—i.e., they would capture noise in our analysis (Ip, 2010). However, a well-fitting model would be preferrable so that, at least, too few specific factors were not chosen. Also note, that the rotation method selected does not rotate the general factor loadings. Thus, the general factor loading pattern is not dependent on the number of retained specific factors.

We used oblique specific factors since it was also considered plausible that specific factors would be correlated. Also, note, that by allowing the specific factors to correlate we also include the possibility of multiple general factors (e.g., internalizing and externalizing) to exist. That is, the correlations of the specific factors could be interpreted as another general factor than the one we explicitly estimate. Absolute differences between orthogonal and oblique specific factor EFAs in the general factor’s standardized factor loadings were all < 0.052, suggesting that the choice between orthogonal and oblique specific factors did not have a large impact. The marginalization procedure, however, accounts for specific factor correlations. As they were small as well, this does not affect the marginalized Fisher information estimates largely. Insomnia and aggression specific factors were moderately negatively correlated correlation *r* = -0.45, *p*-value < 0.01. Otherwise, specific factor correlations were small (|*r|* < 0.15). See Supplementary Table S3 for all specific factor correlations.

## Section 2: Marginalization and Fisher information

We describe the marginalization procedure used here, as it is less commonly used in factor analytic research. A common model used in Item Response Theory (IRT) is the Graded Response Model (GRM; Samejima, 2010) to which a link (reparameterization) from factor models exists. In GRM the essential part is the probability of a response (an answer to some item in our case) conditioned on the latent variable (a general factor in our case). We show one possible way to do the calculations when a factor analysis model is used in parameter estimation. Other variants with additional parameters and different parameterizations are available, and we limit the discussion to how GRM is applied and how marginalization is done in this case.

### Model basics

First, considering when two latent variables $\left( \theta_{1}, \theta_{2} \right)$ load on the item *X* with $K$ ordered categories, the function used to model the probability of observing a response $X=k, k\in1,2,\ldots, K$ is attained followingly:

$P\left( X=1 | \theta_{1},\theta_{2} \right)=\Phi\left( \tau_{1}-\lambda_{1}\theta_{1}-{\lambda_{2}\theta}_{2} \right)$ (1)

which gives the probability for $X = 1$, the first response category. For the last category, $K$

$P\left( X=K | \theta_{1},\theta_{2} \right)=1-\Phi\left( \tau_{K-1}-\lambda_{1}\theta_{1}-{\lambda_{2}\theta}_{2} \right)$

gives the probability, and for arbitrary category $k$

$P\left( X=k | \theta_{1},\theta_{2} \right)- P\left( X=k-1 | \theta_{1},\theta_{2} \right)=\Phi\left( \tau_{k}-\lambda_{1}\theta_{1}-{\lambda_{2}\theta}_{2} \right)-\Phi\left( \tau_{k-1}-\lambda_{1}\theta_{1}-{\lambda_{2}\theta}_{2} \right)$

gives the probability for any response category *m* between the first and the last categories. Other parameterizations exist, but this is the one used here. In above, Φ is the standard normal cumulative distribution function (CDF), $\theta_{1}$ and $\theta_{2}$ are normally distributed latent variables (e.g., the general factor of interest and a specific factor, where the latter is sometimes called a testlet random effect, nuisance, or measurement factors), $\tau$ is the threshold and $\lambda_{1}{, \lambda}_{2}$ are loadings onto the respective latent variables. $\tau$ has $K-1$ finite values, but from here on the subindex is abbreviated. Loadings are equal to $\alpha$ (‘slopes’ commonly in IRT terms), while thresholds are $\tau=slope\times difficulty$ informally put in IRT terms.

Marginalization is needed to make the loadings and thresholds interpretable (and Fisher information computed using them), because a change in $\theta_{1}$ does not cause the same change in probability at different values of $\theta_{2}$. This interrelatedness of the latent variables makes the interpretation of $\lambda_{1}$ hard. A high absolute value of ${\lambda_{2}\theta}_{2}$ makes a change in $\lambda_{1}\theta_{1}$ cause only a small change in probability, whereas the same change would result in a larger change in probability when the absolute value of ${\lambda_{2}\theta}_{2}$ was lower (Φ plateaus at extreme values). If this would be a regular linear model (e.g., a linear regression) we could just hold $\theta_{2}$ constant at some value and interpret how changes in $\theta_{1}$ cause changes in the left-hand side of (1). In our case more work needs to be done, to obtain interpretable parameter values.

### Marginalization

Marginalizing over $\theta_{2}$ means calculating the expectation of the right-hand side of (1) with respect to $\theta_{2}$. Using probability calculus, this calculation of averaging over/integrating out $\theta_{2}$ can be done by using (a slight modification of) the formulas provided at Ip (2010):

$$P\left( X=k | \theta_{1} \right)$$

$$=E_{\theta_{2}}\left[ P\left( X=k | \theta_{1},\theta_{2} \right) \right]$$

$$=E_{\theta_{2}}\left[ \Phi\left( {\tau_{k}-\lambda}_{1}\theta_{1}-{\lambda_{2}\theta}_{2} \right) \right]$$

$$=\int\Phi\left( {\tau_{k}-\lambda}_{1}\theta_{1}-{\lambda_{2}\theta}_{2} \right)\varphi\left( \theta_{2} \right)d\theta_{2}$$

$$=\int P\left( Z \leq{\tau_{k}-\lambda}_{1}\theta_{1}-{\lambda_{2}\theta}_{2}|\theta_{1} \right)\varphi\left( \theta_{2} \right)d\theta_{2}$$

$$=\int P\left( Z+{\lambda_{2}\theta}_{2}\leq{\tau_{k}-\lambda}_{1}\theta_{1}|\theta_{1} \right)\varphi\left( \theta_{2} \right)d\theta_{2}$$

$$=\int\int I\left( Z+{\lambda_{2}\theta}_{2}\leq{\tau_{k}-\lambda}_{1}\theta_{1}|\theta_{1} \right)\varphi\left( \theta_{2} \right)\varphi\left( Z \right)d\theta_{2}dZ$$

$$=E_{{(Z, \theta}_{2})}\left[ I(Z+{\lambda_{2}\theta}_{2}\leq{\tau_{k}-\lambda}_{1}\theta_{1}\left| \theta_{1} \right) \right]$$

$$=P\left( Z+{\lambda_{2}\theta}_{2}\leq{\tau_{k}-\lambda}_{1}\theta_{1} | \theta_{1} \right)$$

Where *Z* is a standard normal variable, $\varphi$ is standard normal density function, $E$ is expectation, and *I* is an indicator function getting the value 1 when the inequality holds and 0 otherwise. Given *Z* was an independent standard normally distributed variable for rewriting Φ, and $\theta_{2}$ was assumed to have standard normal distribution, $Z+{\lambda_{2}\theta}_{2}$ has variance of $Var\left( Z+{\lambda_{2}\theta}_{2} \right)=Var\left( Z \right)+Var\left( {\lambda_{2}\theta}_{2} \right)=1+\lambda_{2}^{2}Var\left( \theta_{2} \right)= 1+\lambda_{2}^{2}$ assuming the common identifiability condition of unit variance for latent variables. Also $E\left( Z \right)=0$ and $E\left( {\lambda_{2}\theta}_{2} \right)=\lambda_{2}E\left( \theta_{2} \right)=0$. As sums of normally distributed variables are again normally distributed, we have:

$\left( Z+{\lambda_{2}\theta}_{2} \right)\sim N\left( 0, 1+\lambda_{2}^{2} \right)$.

Now, if we standardize $Z+{\lambda_{2}\theta}_{2}$ we get the inequality within $P\left( X=k | \theta_{1} \right)$ modified as

$$\frac{Z+{\lambda_{2}\theta}_{2}}{\sqrt{1+\lambda_{2}^{2}}}\leq\frac{{\tau_{k}-\lambda}_{1}\theta_{1}}{\sqrt{1+\lambda_{2}^{2}}}=\frac{\tau_{k}}{\sqrt{1+\lambda_{2}^{2}}}-\frac{\lambda_{1}}{\sqrt{1+\lambda_{2}^{2}}}\theta_{1}= \tau_{k, marg}-\lambda_{1, marg}\theta_{1},$$

meaning that the left-hand side of the inequality is again a standard normal variable, and we can compute the marginal probability with respect to $\theta_{1}$:

$$P\left( X=1 | \theta_{1} \right)=\Phi\left( \tau_{k,marg}-\lambda_{1, marg}\theta_{1} \right),$$

for the first category, where $\tau_{k,marg}=\frac{\tau_{k}}{\sqrt{1+\lambda_{2}^{2}}}$ and $\lambda_{1, marg}=\frac{\lambda_{1}}{\sqrt{1+\lambda_{2}^{2}}}$.

Using the marginalized parameters, we can calculate the probabilities for any response category with the same logic. In effect, the marginalization procedure rescales the model by 1 + the squared loading on the specific latent variable.

Marginalization generalizes to multiple specific variables as well.

$P\left( Z+\boldsymbol{\lambda}_{s}^{'}\boldsymbol{\lambda}_{s}\leq{\tau_{k}-\lambda}_{1}\theta_{1}|\theta_{1} \right)$,

where $\boldsymbol{\lambda}_{s}^{'}\boldsymbol{\theta}_{s}$ the dot product of vectors of specific factor loadings as well as specific factors $s\in1, 2, 3, \ldots S$, which we want to average over (integrate out). Applying the same treatment as above we get

$$\frac{Z+\boldsymbol{\lambda}_{s}^{'}\boldsymbol{\lambda}_{s}}{\sqrt{1+\boldsymbol{\lambda}_{s}^{'}\boldsymbol{\lambda}_{s}}}\leq\frac{{\tau_{m}-\lambda}_{1}\theta_{1}}{\sqrt{1+\boldsymbol{\lambda}_{s}^{'}\boldsymbol{\lambda}_{s}}}=\frac{\tau_{m}}{\sqrt{1+\boldsymbol{\lambda}_{s}^{'}\boldsymbol{\lambda}_{s}}}-\frac{\lambda_{1}}{\sqrt{1+\boldsymbol{\lambda}_{s}^{'}\boldsymbol{\lambda}_{s}}}\theta_{1}= \tau_{m,marg}-\lambda_{1, marg}\theta_{1}$$

Further, for correlated specific factors, with a correlation matrix $\boldsymbol{\Psi}$,

$$\frac{\tau_{m}}{\sqrt{1+\boldsymbol{\lambda}_{s}^{'}\boldsymbol{\Psi}\boldsymbol{\lambda}_{\boldsymbol{s}}}}-\frac{\lambda_{1}}{\sqrt{1+\boldsymbol{\lambda}_{\boldsymbol{s}}^{\mathbf{'}}\boldsymbol{\Psi}\boldsymbol{\lambda}_{\boldsymbol{s}}}}\theta_{1}= \tau_{m,marg}-\lambda_{1, marg}\theta_{1}$$

Again, this needs to be applied separately to other categories as well.

If specific factors are positively correlated, the marginalizing leads to smaller marginal parameters. If specific factors are negatively correlated, the opposite happens.

### Fisher information

For completeness, Fisher Information (for a single item, subscript omitted) is defined as the variance of the ‘score’ function

$$I\left( \theta_{1} \right)=E_{X|\theta_{1}}\left[ \left( \frac{\partial}{\partial\theta_{1}}\log P\left( X | \theta_{1} \right) \right)^{2} \right]$$

$$=E_{X|\theta_{1}}\left[ \left( \frac{\frac{\partial}{\partial\theta_{1}}P\left( X | \theta_{1} \right)}{P\left( X | \theta_{1} \right)} \right)^{2} \right]$$

$$=\sum_{m=1}^{k} \frac{\left( \frac{\partial}{\partial\theta_{1}}P\left( X=m | \theta_{1} \right) \right)^{2}}{P\left( X=m | \theta_{1} \right)}.$$

It is typically (for example, in *Mplus* -software; see <https://www.statmodel.com/download/MplusIRT.pdf>) computed by using the logit model (not shown here) and then applying a logit to probit approximation (multiplication by a certain constant), as it is simpler. Marginalized loadings and thresholds are used when evaluating the probabilities in equations above.

From the above expression, looking at the numerator, we see that the information grows as there are larger rates of change in probability of some response $m$ at the value of the factor. E.g., if there is a rapidly growing chance that a person reports suicidal thoughts when $1.5<\theta_{1}<2$, then at this value of the factor the suicidal thought report is informative of it. If at lower levels of $-3<\theta_{1}<0$ the probability of a person reporting suicidal thoughts is consistently low, and so does not change much, then there is less information on the factor at these values. Fisher information should not be mistaken for Shannon information, also known as entropy, or its generalizations.

## Section 3: R -script for bi-factor marginalization

R -script below can be used for computing marginalized information from a lavaan fit.

Note that the script will automatically install dependencies (R -packages).

# Lavaan to marginalized IRT example.

# Fetch necessary functions from a GitHub repository.

source**(**"https://raw.githubusercontent.com/lintula9/Lav2IRT/main/L2IRT_stable.R"**)**

# Example 1, bifactor model simulated data.

TrueModel **<-** "

# General factor

p =~ .6 * X1 + .6 * X2 + .6 * X3 + .6 * X4 + .6 * X5 + .6 * X6

# Nuisance factor

nuisance =~ .8 * X1 + .3 * X2 + .1 * X3

X1 | -1*t1 + 0*t2 + 1*t3

X2 | -1.5*t1 + 0*t2 + 1.5*t3

X3 | -2*t1 + 0*t2 + 2*t3

X4 | -1*t1 + 0*t2 + 1*t3

X5 | -1*t1 + 0*t2 + 1*t3

X6 | -1*t1 + 0*t2 + 1*t3

p ~~ 0*nuisance

nuisance ~~ 1*nuisance

p ~~ 1*p "

X **<-** data.frame**(**lavaan**::**simulateData**(**TrueModel, model.type **=** F,

int.lv.free **=** F,

std.lv **=** T,

sample.nobs **=** 10000, orthogonal **=** T,

standardized **=** T**))**

lavaanresult **=** lavaan**::**efa**(**X,

estimator **=** "WLSMV",

parameterization **=** "theta", # Required

rotation **=** "bigeomin",

std.lv **=** T, # Required

ordered **=** T,

mimic **=** "mplus", # Required

nfactors **=** 2,

rotation.args **=** list**(**orthogonal **=** T**))$**nf2

# One item

Probs **=** LavaanIRTProbabilities**(**lavaanfit **=** lavaanresult,

varname **=** "X1",

dimname **=** "f2",

dimmin **=** **-**9,

dimmax **=** 9,

marginalize **=** T,

std **=** F #Default, F, is required for marginalization.

**)**

ItemInfos **=** ItemInformation**(**Probs**)**

# For plotting you might want to translate informations to numeric using as.numeric(ItemInfos), for example.

plot**(**as.numeric**(**ItemInfos**)**, type **=** "l", xlab **=** expression**(**"Factor" **~** "score" **~** theta**)**, ylab **=** expression**(**I**(**theta**))**, family **=** "serif" **)**

# Requires ggplot2 package.

ggplot2**::**ggplot**()** **+**

geom_line**(**aes**(**y **=** as.numeric**(**ItemInfos**)**, # manual switch to numeric class is currently required.

x **=** seq**(-**9, 9, length.out **=** length**(**ItemInfos**))))**

# Multiple items

setinfos **=** SetInformation**(**lavaanresult,

itemset **=** c**(**"X1", "X2"**)**, points **=** seq**(-**9,9,by **=** 0.01**)**, dimname **=** "f2", marginalize **=** T, std **=** F**)**

setinfos

## Section 4: Supplementary tables

| Supplementary Table S1.  *General Factor parameters and total item information.* | | | | | | | | | | | |
| --- | --- | --- | --- | --- | --- | --- | --- | --- | --- | --- | --- |
| Item |  | General Factor loadings^a,b^ | | |  | Marginal Thresholds | | | |  |  |
|  |  | Raw λ | Marginal λ | Marginal λ 95% CI |  | $\tau_{1}$ | $\tau_{2}$ | $\tau_{3}$ | $\tau_{4}$ |  | Total Item information |
| CORE-OM 1 |  | 1.05 | 0.87 | (0.81, 0.97) |  | -1.54 | -0.53 | 0.57 | 1.83 |  | 4.48 |
| CORE-OM 2¹⁰ |  | 1.34 | 1.22 | (0.93, 1.28) |  | -3.22 | -1.55 | -0.12 | 1.81 |  | 7.55 |
| CORE-OM 3¹⁰ |  | 0.47 | 0.36 | (0.32, 0.50) |  | -0.81 | 0.13 | 0.97 | 1.96 |  | 1.68 |
| CORE-OM 4 |  | 1.27 | 0.85 | (0.78, 1.12) |  | -2.46 | -1.19 | 0.24 | 1.75 |  | 4.89 |
| CORE-OM 5 |  | 1.40 | 1.11 | (1.06, 1.39) |  | -1.74 | -0.43 | 0.72 | 2.15 |  | 6.15 |
| CORE-OM 6 |  | 0.35 | 0.21 | (0.16, 0.35) |  | 1.80 | 2.47 | 3.03 | 3.44 |  | 0.77 |
| CORE-OM 7¹⁰ |  | 0.89 | 0.76 | (0.70, 0.92) |  | -1.57 | -0.24 | 1.08 | 2.39 |  | 4.25 |
| CORE-OM 8 |  | 0.33 | 0.31 | (0.28, 0.37) |  | -0.99 | -0.27 | 0.30 | 1.07 |  | 1.25 |
| CORE-OM 9 |  | 1.74 | 0.79 | (0.72, 0.87) |  | 0.84 | 1.63 | 2.27 | 3.21 |  | 3.43 |
| CORE-OM 10¹⁰ |  | 1.03 | 0.87 | (0.84, 1.05) |  | -1.07 | 0.08 | 1.18 | 2.33 |  | 4.53 |
| CORE-OM 11 |  | 1.20 | 0.91 | (0.84, 1.03) |  | -0.74 | 0.18 | 1.08 | 2.28 |  | 4.43 |
| CORE-OM 12 |  | 1.21 | 0.81 | (0.74, 1.12) |  | -2.09 | -0.70 | 0.73 | 2.26 |  | 4.74 |
| CORE-OM 13 |  | 1.39 | 1.26 | (0.85, 1.31) |  | -2.79 | -1.26 | 0.01 | 1.82 |  | 7.53 |
| CORE-OM 14 |  | 0.77 | 0.74 | (0.57, 0.78) |  | -0.98 | 0.02 | 0.96 | 2.36 |  | 3.77 |
| CORE-OM 15¹⁰ |  | 1.03 | 0.88 | (0.62, 0.95) |  | -0.08 | 0.85 | 1.78 | 2.91 |  | 4.28 |
| CORE-OM 16¹⁰ |  | 1.87 | 0.81 | (0.72, 0.90) |  | 1.52 | 2.29 | 2.84 | 3.64 |  | 3.30 |
| CORE-OM 17 |  | 1.73 | 1.56 | (1.24, 1.70) |  | -1.69 | -0.08 | 1.26 | 2.79 |  | 9.21 |
| CORE-OM 18¹⁰ |  | 0.63 | 0.38 | (0.36, 0.48) |  | -1.19 | -0.44 | 0.16 | 0.94 |  | 1.55 |
| CORE-OM 19 |  | 0.32 | 0.27 | (0.22, 0.41) |  | -0.76 | 0.16 | 0.83 | 1.53 |  | 1.13 |
| CORE-OM 20 |  | 1.05 | 0.99 | (0.75, 1.05) |  | -2.30 | -0.97 | 0.07 | 1.38 |  | 5.35 |
| CORE-OM 21 |  | 0.76 | 0.55 | (0.52, 0.79) |  | -0.92 | 0.28 | 1.18 | 2.47 |  | 2.83 |
| CORE-OM 22 |  | 0.34 | 0.23 | (0.18, 0.34) |  | 1.49 | 2.13 | 2.72 |  |  | 0.73 |
| CORE-OM 23¹⁰ |  | 1.95 | 1.75 | (1.41, 1.91) |  | -1.80 | -0.12 | 1.25 | 3.02 |  | 10.67 |
| CORE-OM 24 |  | 1.85 | 1.00 | (0.92, 1.09) |  | 0.48 | 1.35 | 2.05 | 2.81 |  | 4.28 |
| CORE-OM 25 |  | 0.71 | 0.51 | (0.46, 0.59) |  | -0.87 | 0.01 | 0.83 | 1.84 |  | 2.35 |
| CORE-OM 26 |  | 0.68 | 0.48 | (0.45, 0.62) |  | -0.24 | 0.40 | 1.02 | 1.73 |  | 1.90 |
| CORE-OM 27¹⁰ |  | 1.75 | 1.53 | (1.35, 1.66) |  | -2.08 | -0.49 | 0.86 | 2.49 |  | 9.15 |
| CORE-OM 28¹⁰ |  | 0.96 | 0.89 | (0.64, 0.94) |  | -1.88 | -0.69 | 0.30 | 1.65 |  | 4.69 |
| CORE-OM 29 |  | 0.60 | 0.53 | (0.48, 0.60) |  | -1.04 | 0.03 | 1.00 | 2.19 |  | 2.67 |
| CORE-OM 30 |  | 0.89 | 0.76 | (0.69, 0.82) |  | -1.51 | -0.62 | 0.24 | 1.37 |  | 3.60 |
| CORE-OM 31 |  | 1.02 | 0.78 | (0.71, 1.03) |  | -1.88 | -0.77 | 0.43 | 1.72 |  | 4.17 |
| CORE-OM 32 |  | 0.90 | 0.67 | (0.61, 0.92) |  | -1.99 | -0.81 | 0.36 | 1.60 |  | 3.55 |
| CORE-OM 33 |  | 0.66 | 0.49 | (0.42, 0.57) |  | 0.39 | 1.12 | 1.80 | 2.70 |  | 2.06 |
| CORE-OM 34 |  | 0.63 | 0.49 | (0.43, 0.59) |  | 1.79 | 2.36 | 2.80 | 3.30 |  | 1.70 |
| OASIS 1 |  | 1.51 | 1.20 | (0.78, 1.25) |  | -3.03 | -1.58 | 0.06 | 2.23 |  | 7.53 |
| OASIS 2 |  | 1.28 | 1.00 | (0.69, 1.04) |  | -2.87 | -1.06 | 0.94 | 2.83 |  | 6.52 |
| OASIS 3 |  | 0.88 | 0.63 | (0.57, 0.69) |  | -1.28 | -0.32 | 0.76 | 2.03 |  | 3.23 |
| OASIS 4 |  | 1.20 | 0.84 | (0.75, 0.94) |  | -1.40 | -0.08 | 1.20 | 2.29 |  | 4.55 |
| OASIS 5 |  | 1.13 | 0.81 | (0.74, 0.88) |  | -1.45 | -0.17 | 1.03 | 2.17 |  | 4.36 |
| PHQ-9 1 |  | 1.26 | 0.94 | (0.87, 1.29) |  | -0.93 | 0.79 | 1.49 |  |  | 3.80 |
| PHQ-9 2 |  | 1.77 | 1.42 | (1.30, 1.69) |  | -1.59 | 0.96 | 1.85 |  |  | 6.40 |
| PHQ-9 3 |  | 1.48 | 0.45 | (0.42, 0.61) |  | -0.94 | 0.22 | 0.71 |  |  | 1.57 |
| PHQ-9 4 |  | 0.94 | 0.69 | (0.65, 0.96) |  | -1.75 | 0.01 | 0.63 |  |  | 2.76 |
| PHQ-9 5 |  | 0.62 | 0.53 | (0.50, 0.71) |  | -0.21 | 0.76 | 1.29 |  |  | 1.79 |
| PHQ-9 6 |  | 1.34 | 0.97 | (0.90, 1.19) |  | -1.32 | 0.32 | 1.12 |  |  | 3.99 |
| PHQ-9 7 |  | 0.64 | 0.57 | (0.55, 0.71) |  | -0.59 | 0.57 | 1.13 |  |  | 2.05 |
| PHQ-9 8 |  | 0.54 | 0.49 | (0.46, 0.60) |  | 0.65 | 1.59 | 2.11 |  |  | 1.65 |
| PHQ-9 9 |  | 2.07 | 0.87 | (0.79, 0.98) |  | 0.90 | 2.13 | 2.60 |  |  | 3.10 |
| AUDIT 1 |  | -0.02 | -0.02 | (-0.06, 0.04) |  | -0.77 | 0.48 | 1.52 | 2.48 |  | 0.09 |
| AUDIT 2 |  | 0.03 | 0.03 | (-0.02, 0.10) |  | 0.36 | 0.96 | 1.51 | 2.05 |  | 0.10 |
| n = 5223  WLSMV Estimator. | | | | | | | | | | | |
| All general factor loadings were significant at p < 0.05, except for AUDIT-1 and AUDIT-2. | | | | | | | | | | | |
| ^b^Raw λ are ordinary unstandardized factor loadings. Marginal λ are unstandardized, marginalized loadings. | | | | | | | | | | | |
| ^10^CORE-10 -item. | | | | | | | | | | | |
|  | | | | | | | | | | | |

| Supplementary Table S2.  *All standardized (unmarginalized) factor loadings of exploratory factor analysis with 9 factors.* | | | | | | | | | | | | |  |
| --- | --- | --- | --- | --- | --- | --- | --- | --- | --- | --- | --- | --- | --- |
| Item |  | Factor | | | | | | | | | |  | |
|  |  | General | Anxiety | | Sense of nonaccomplishment | Somatic pain | Aggression | Loneliness | Insomnia | Suicidality | Sense of accomplishment |  | |
| CORE-OM 1 |  | **0.66** | | -0.07 | 0.01 | 0.06 | -0.02 | **0.41** | 0.02 | 0.00 | 0.01 |  | |
| CORE-OM 2 |  | **0.77** | | 0.07 | 0.05 | 0.09 | 0.11 | -0.06 | 0.02 | -0.18 | -0.07 |  | |
| CORE-OM 3 |  | **0.34** | | -0.07 | -0.01 | -0.01 | 0.01 | **0.47** | 0.05 | 0.04 | **0.31** |  | |
| CORE-OM 4 |  | **0.65** | | -0.01 | **0.42** | -0.01 | -0.21 | 0.01 | 0.02 | -0.01 | **0.31** |  | |
| CORE-OM 5 |  | **0.74** | | 0.00 | -0.13 | 0.29 | -0.02 | -0.01 | -0.10 | -0.06 | 0.15 |  | |
| CORE-OM 6 |  | 0.21 | | -0.01 | -0.01 | 0.00 | **0.75** | -0.03 | -0.29 | 0.01 | 0.23 |  | |
| CORE-OM 7 |  | **0.60** | | 0.05 | 0.08 | -0.08 | -0.01 | 0.00 | 0.02 | 0.08 | **0.39** |  | |
| CORE-OM 8 |  | **0.30** | | 0.01 | -0.15 | **0.33** | 0.11 | 0.00 | 0.10 | -0.02 | -0.03 |  | |
| CORE-OM 9 |  | **0.62** | | 0.01 | 0.00 | -0.01 | 0.12 | -0.05 | -0.01 | **0.70** | -0.08 |  | |
| CORE-OM 10 |  | **0.66** | | 0.22 | -0.03 | 0.18 | 0.04 | 0.17 | -0.10 | -0.02 | 0.09 |  | |
| CORE-OM 11 |  | **0.67** | | **0.44** | -0.07 | 0.02 | 0.08 | 0.07 | -0.01 | 0.01 | 0.16 |  | |
| CORE-OM 12 |  | **0.63** | | 0.02 | **0.37** | -0.02 | -0.16 | -0.02 | -0.01 | -0.05 | **0.42** |  | |
| CORE-OM 13 |  | **0.78** | | -0.02 | 0.00 | -0.04 | 0.03 | -0.03 | 0.20 | -0.04 | -0.08 |  | |
| CORE-OM 14 |  | **0.59** | | -0.08 | -0.04 | 0.01 | 0.15 | -0.03 | 0.05 | -0.04 | -0.07 |  | |
| CORE-OM 15 |  | **0.66** | | 0.14 | -0.17 | -0.06 | 0.27 | 0.00 | 0.09 | -0.01 | 0.00 |  | |
| CORE-OM 16 |  | **0.63** | | 0.00 | -0.07 | -0.05 | 0.14 | 0.01 | 0.01 | **0.70** | 0.00 |  | |
| CORE-OM 17 |  | **0.84** | | -0.01 | -0.12 | -0.05 | 0.08 | -0.02 | 0.02 | 0.05 | 0.19 |  | |
| CORE-OM 18 |  | **0.35** | | -0.04 | -0.03 | **0.68** | 0.01 | 0.02 | **0.64** | -0.01 | 0.00 |  | |
| CORE-OM 19 |  | 0.26 | | 0.00 | -0.01 | 0.03 | -0.07 | 0.28 | 0.01 | 0.14 | **0.36** |  | |
| CORE-OM 20 |  | **0.70** | | -0.05 | -0.01 | 0.01 | 0.01 | -0.05 | 0.17 | -0.12 | 0.02 |  | |
| CORE-OM 21 |  | **0.48** | | 0.25 | 0.00 | 0.15 | 0.03 | -0.04 | -0.06 | -0.02 | **0.50** |  | |
| CORE-OM 22 |  | 0.23 | | -0.03 | 0.01 | 0.00 | **0.68** | 0.00 | -0.28 | 0.03 | 0.11 |  | |
| CORE-OM 23 |  | **0.87** | | -0.11 | -0.02 | -0.05 | 0.04 | 0.01 | -0.01 | 0.07 | 0.16 |  | |
| CORE-OM 24 |  | **0.71** | | -0.07 | 0.02 | -0.01 | 0.00 | 0.02 | -0.02 | **0.58** | -0.01 |  | |
| CORE-OM 25 |  | **0.45** | | 0.02 | **0.38** | 0.00 | **0.35** | 0.29 | -0.01 | -0.10 | -0.04 |  | |
| CORE-OM 26 |  | **0.44** | | -0.04 | 0.03 | 0.01 | 0.02 | **0.62** | -0.03 | 0.02 | 0.02 |  | |
| CORE-OM 27 |  | **0.84** | | -0.16 | -0.06 | 0.02 | -0.02 | 0.15 | -0.01 | 0.00 | 0.11 |  | |
| CORE-OM 28 |  | **0.66** | | 0.04 | 0.03 | -0.02 | 0.10 | 0.06 | 0.22 | -0.02 | -0.07 |  | |
| CORE-OM 29 |  | **0.47** | | -0.03 | 0.18 | 0.11 | **0.30** | 0.03 | -0.09 | -0.21 | -0.01 |  | |
| CORE-OM 30 |  | **0.60** | | -0.05 | **0.41** | -0.04 | 0.00 | 0.03 | 0.03 | 0.02 | 0.00 |  | |
| CORE-OM 31 |  | **0.62** | | -0.06 | 0.07 | -0.01 | -0.14 | 0.04 | 0.01 | 0.10 | **0.43** |  | |
| CORE-OM 32 |  | **0.55** | | 0.08 | 0.15 | 0.02 | -0.07 | 0.01 | -0.01 | -0.01 | **0.51** |  | |
| CORE-OM 33 |  | **0.44** | | 0.05 | 0.16 | -0.02 | **0.48** | **0.34** | 0.03 | -0.01 | 0.01 |  | |
| CORE-OM 34 |  | **0.44** | | 0.13 | 0.00 | 0.05 | 0.29 | 0.05 | 0.01 | **0.48** | 0.01 |  | |
|  |  |  | |  |  |  |  |  |  |  |  |  | |
| PHQ-9 1 |  | **0.68** | | 0.02 | 0.01 | **0.31** | -0.21 | 0.04 | -0.12 | 0.02 | 0.11 |  | |
| PHQ-9 2 |  | **0.82** | | -0.09 | -0.03 | 0.20 | -0.21 | 0.01 | -0.06 | 0.07 | 0.01 |  | |
| PHQ-9 3 |  | **0.41** | | 0.00 | 0.01 | **0.83** | -0.10 | -0.01 | **0.67** | 0.04 | 0.01 |  | |
| PHQ-9 4 |  | **0.57** | | 0.00 | 0.01 | **0.56** | -0.12 | -0.07 | 0.00 | -0.09 | -0.01 |  | |
| PHQ-9 5 |  | **0.47** | | 0.11 | 0.11 | **0.40** | 0.00 | -0.01 | 0.01 | 0.08 | -0.02 |  | |
| PHQ-9 6 |  | **0.70** | | -0.03 | **0.45** | 0.10 | -0.14 | -0.01 | -0.04 | 0.02 | 0.00 |  | |
| PHQ-9 7 |  | **0.50** | | 0.16 | 0.11 | **0.31** | 0.02 | -0.02 | 0.00 | 0.00 | 0.03 |  | |
| PHQ-9 8 |  | **0.44** | | 0.21 | 0.02 | 0.25 | 0.19 | 0.02 | -0.01 | 0.03 | -0.01 |  | |
| PHQ-9 9 |  | **0.66** | | -0.04 | 0.02 | 0.06 | -0.05 | -0.02 | -0.03 | **0.67** | -0.09 |  | |
|  |  |  | |  |  |  |  |  |  |  |  |  | |
| OASIS 1 |  | **0.77** | | 0.27 | 0.00 | -0.02 | -0.03 | -0.08 | 0.14 | -0.10 | -0.13 |  | |
| OASIS 2 |  | **0.71** | | **0.38** | -0.04 | -0.06 | 0.00 | -0.06 | 0.12 | 0.02 | -0.09 |  | |
| OASIS 3 |  | **0.54** | | **0.56** | 0.06 | 0.00 | -0.02 | 0.15 | 0.02 | 0.04 | 0.01 |  | |
| OASIS 4 |  | **0.64** | | **0.54** | 0.00 | -0.01 | -0.03 | 0.02 | 0.01 | 0.00 | 0.10 |  | |
| OASIS 5 |  | **0.63** | | **0.47** | 0.05 | 0.01 | 0.00 | 0.23 | -0.01 | -0.01 | -0.03 |  | |
|  |  |  | |  |  |  |  |  |  |  |  |  | |
| AUDIT 1 |  | -0.02 | | -0.09 | 0.26 | 0.01 | 0.06 | -0.20 | 0.08 | 0.02 | 0.06 |  | |
| AUDIT 2 |  | 0.03 | | 0.00 | **0.33** | 0.03 | 0.03 | -0.19 | 0.10 | 0.12 | 0.05 |  | |
| Sum of Squares |  | 17.6 | | 2.2 | 2.0 | 1.9 | 1.7 | 1.6 | 1.3 | 1.3 | 0.9 |  | |
| Prop. Explained |  | 0.576 | | 0.072 | 0.065 | 0.064 | 0.056 | 0.052 | 0.044 | 0.041 | 0.031 |  | |
| Standardized factor loadings > 0.300 in absolute value are in bold.  RMSEA 0.045 (95% CI (0.044, 0.046))  SRMR 0.024  CFI 0.972  TLI 0.958 | | | | | | | | | | | | |  |

| Supplementary Table S3.  *Factor correlations in explorative factor analysis.* | | | | | | | | | |
| --- | --- | --- | --- | --- | --- | --- | --- | --- | --- |
|  | General | Anxiety | Sense of NA* | Somatic pain | Aggression | Loneliness | Insomnia | Suicidality | Sense of accomplishment |
| General | 1.00 | 0.00 | 0.00 | 0.00 | 0.00 | 0.00 | 0.00 | 0.00 | 0.00 |
| F2 | 0.00 | 1.00 | 0.03 | 0.05 | 0.00 | 0.02 | 0.07 | -0.09 | -0.02 |
| F3 | 0.00 | 0.03 | 1.00 | 0.13 | 0.07 | 0.13 | -0.11 | 0.17 | 0.01 |
| F4 | 0.00 | 0.05 | 0.13 | 1.00 | -0.01 | 0.08 | -0.35 | 0.11 | 0.22 |
| F5 | 0.00 | 0.00 | 0.07 | -0.01 | 1.00 | -0.07 | 0.12 | -0.01 | -0.14 |
| F6 | 0.00 | 0.02 | 0.13 | 0.08 | -0.07 | 1.00 | -0.05 | 0.08 | 0.11 |
| F7 | 0.00 | 0.07 | -0.11 | -0.35 | 0.12 | -0.05 | 1.00 | -0.12 | -0.08 |
| F8 | 0.00 | -0.09 | 0.17 | 0.11 | -0.01 | 0.08 | -0.12 | 1.00 | 0.18 |
| F9 | 0.00 | -0.02 | 0.01 | 0.22 | -0.14 | 0.11 | -0.08 | 0.18 | 1.00 |
| * Non-accomplishment | | | | | | | | | |

| Supplementary Table S4.  *Longitudinal measurement invariance testing for CORE-10, PHQ-9 & OASIS.* | | | | | | | |
| --- | --- | --- | --- | --- | --- | --- | --- |
|  | CFI | TLI | RMSEA | RMSEA 95% CI | | SRMR | ΔCFI |
|  | CORE-10 | | | | | | |
| Configural | 0.985 | 0.982 | 0.043 | 0.046 | 0.040 | 0.036 |  |
| Loadings | 0.987 | 0.985 | 0.039 | 0.042 | 0.037 | 0.037 | 0.002 |
| Thresholds | 0.984 | 0.984 | 0.041 | 0.043 | 0.038 | 0.036 | -0.003 |
| Residuals | 0.985 | 0.985 | 0.039 | 0.041 | 0.036 | 0.037 | 0.001 |
|  | OASIS | | | | | | |
| Configural | 0.980 | 0.969 | 0.102 | 0.109 | 0.096 | 0.039 |  |
| Loadings | 0.982 | 0.976 | 0.091 | 0.097 | 0.085 | 0.040 | 0.002 |
| Thresholds | 0.980 | 0.979 | 0.086 | 0.091 | 0.081 | 0.040 | -0.002 |
| Residuals | 0.980 | 0.981 | 0.081 | 0.086 | 0.076 | 0.040 | 0.000 |
|  | PHQ-9 | | | | | | |
|  |  |  |  |  |  |  |  |
| Configural | 0.969 | 0.962 | 0.062 | 0.065 | 0.059 | 0.050 |  |
| Loadings | 0.972 | 0.968 | 0.056 | 0.059 | 0.053 | 0.050 | 0.003 |
| Thresholds | 0.968 | 0.966 | 0.058 | 0.061 | 0.056 | 0.050 | -0.004 |
| Residuals | 0.971 | 0.971 | 0.054 | 0.057 | 0.051 | 0.050 | 0.003 |
| WLSMV estimator  All indices are adjusted for the estimator. | | | | | | | |
| n = 3099  Note that loadings invariance model must fit as well as or better as the configural model, when using WLSMV.  CORE-OM and AUDIT-C were not included, as previous research has analysed the former and the latter was not a feasible measure of a general factor. | | | | | | | |

## Section 5: Sensitivity analysis omitting CORE-OM items not also included in CORE-10

For the sensitivity analysis omitting CORE-OM items, bifactor rotation with 9 oblique factors was conducted. The total information was 52.0 for CORE-10, 31.0 for PHQ-9 and 25.3 for OASIS. After dividing by length, the total information was 5.2, 3.4 and 5.1, respectively. The total item information estimates are presented in a table below.

| *Supplementary table S5.* Total item information when omitting CORE-OM items not included in CORE-10. | | |
| --- | --- | --- |
| Item |  | Total item information |
| CORE-OM 23 |  | 10.0 |
| CORE-OM 27 |  | 10.0 |
| PHQ-9 2 |  | 7.20 |
| CORE-OM 2 |  | 6.31 |
| OASIS 1 |  | 6.23 |
| OASIS 2 |  | 5.76 |
| CORE-OM 10 |  | 5.50 |
| OASIS 5 |  | 4.93 |
| OASIS 4 |  | 4.82 |
| CORE-OM 7 |  | 4.82 |
| PHQ-9 1 |  | 4.78 |
| PHQ-9 6 |  | 4.10 |
| CORE-OM 28 |  | 4.06 |
| CORE-OM 15 |  | 3.79 |
| OASIS 3 |  | 3.59 |
| CORE-OM 16 |  | 3.50 |
| PHQ-9 9 |  | 3.18 |
| PHQ-9 4 |  | 3.09 |
| PHQ-9 7 |  | 2.38 |
| CORE-OM 3 |  | 2.26 |
| PHQ-9 5 |  | 2.08 |
| PHQ-9 3 |  | 1.97 |
| PHQ-9 8 |  | 1.93 |
| CORE-OM 18 |  | 1.78 |

## References

Horn, J. L. (1965). A rationale and test for the number of factors in factor analysis. *Psychometrika*, *30*(2), 179–185. https://doi.org/10.1007/BF02289447

Ip, E. H. (2010). Empirically indistinguishable multidimensional IRT and locally dependent unidimensional item response models. *British Journal of Mathematical and Statistical Psychology*, *63*(2), 395–416. https://doi.org/10.1348/000711009X466835

Jennrich, R. I., & Bentler, P. M. (2012). Exploratory Bi-factor Analysis: The Oblique Case. *Psychometrika*, *77*(3), 442–454. https://doi.org/10.1007/s11336-012-9269-1

Lim, S., & Jahng, S. (2019). Determining the Number of Factors Using Parallel Analysis and Its Recent Variants. *Psychological Methods*, *24*. https://doi.org/10.1037/met0000230

Rosseel, Y. (2012). lavaan: An R Package for Structural Equation Modeling. *Journal of Statistical Software*, *48*, 1–36. https://doi.org/10.18637/jss.v048.i02

Samejima, F. (2010). The General Graded Response Model. In *Handbook of Polytomous Item Response Theory Models*. Routledge.
